# Supplementary material for: Genetic diversity, relatedness and inbreeding of ranched and fragmented Cape buffalo populations in southern Africa
Source: PLoS One. 2020 Aug 14;15(8):e0236717. doi: 10.1371/journal.pone.0236717 (PMC7428177; doi:10.1371/journal.pone.0236717)
Supplement: S1 Appendix — (PDF) [file pone.0236717.s001.pdf]

S1 Appendix. Supplementary methods

Genetic diversity, relatedness and inbreeding of ranched and fragmented Cape buffalo populations in southern Africa

Deon de Jager, Cindy Kim Harper, Paulette Bloomer

Population differentiation

To determine the number of gene pools that may be contributing to buffalo populations on the private ranches we performed Bayesian clustering of microsatellite genotypes using STRUCTURE v2.3.4 [1]. We used a burn-in of 100,000 and 500,000 MCMC repetitions for *K* values from one to nine, with no location priors, using the admixture and correlated allele frequency models. The optimum *K* (number of different genetic clusters) was determined using the Evanno method [2] implemented in Structure Harvester [3] (http://taylor0.biology.ucla.edu/structureHarvester/), in conjunction with the likelihood of *K* (Ln Pr(*X*|*K*). The CLUMPAK Main Pipeline [4] (http://clumpak.tau.ac.il/index.html), with default parameters, was used to assign individuals to a group and to create the graphical output. Discriminant analysis of principal components (DAPC) [5] was conducted at the optimum *K* using the package adegenet v2.1.1 [6] in R v3.5.0 [7]. Rstudio v1.2.5033 [8] was used for all R-based analyses in this manuscript.

Additional structure analyses were done where relatives were removed. The reasons for this were two-fold: First, the presence of close relatives in the data set, as would be the case with African buffalo given their social hierarchy and breeding strategy, can affect the ability of Bayesian clustering algorithms, as implemented in STRUCTURE, to accurately identify the number of subpopulations [9]. Second, the model implemented in STRUCTURE assumes unlinked loci in linkage equilibrium and Hardy-Weinberg equilibrium (HWE) within populations [1]. Both the

hierarchical breeding strategy in unmanaged buffalo herds, as well as intensive breeding practices on private ranches may cause deviations from these assumptions by way of a higher proportion of close relatives than expected by chance. We postulated that removing close relatives would provide us with a subset of samples from each locality that would conform to HWE.

Relatives were thus removed using the TrioML relatedness estimates, obtained before, in conjunction with the software Friends and Family v22 [10] at a relatedness cut-off value of 0.25. See S7 Table for sample sizes before and after removal of relatives. Linkage disequilibrium and HWE exact tests for both the full data set and the relatives removed data set were carried out in GenePop v4.7.0 [11], using 10,000 dememorizations, 1,000 batches and 5,000 iterations per batch. Complete enumeration of loci was carried out when applicable. The significance level (*p*-value) for each test was Bonferroni-adjusted for multiple testing [12], using the standard Bonferroni method, in R v3.5.0. The STRUCTURE analysis of the relatives removed data set was carried out with the same parameters as with the full data set.

References for supplementary methods

1. Pritchard JK, Stephens M, Donnelly P. Inference of population structure using multilocus genotype data. Genetics. 2000;155(2):945-59. PubMed PMID: PMC1461096.
2. Evanno G, Regnaut S, Goudet J. Detecting the number of clusters of individuals using the software structure: A simulation study. Mol Ecol. 2005;14(8):2611-20. doi: 10.1111/j.1365-294X.2005.02553.x.
3. Earl DA, vonHoldt BM. STRUCTURE HARVESTER: A website and program for visualizing STRUCTURE output and implementing the Evanno method. Conserv Genet Resour. 2012;4(2):359-61. doi: 10.1007/s12686-011-9548-7.
4. Kopelman NM, Mayzel J, Jakobsson M, Rosenberg NA, Mayrose I. CLUMPAK: A program for identifying clustering modes and packaging population structure inferences across K. Mol Ecol Resour. 2015;15(5):1179-91. doi: 10.1111/1755-0998.12387. PubMed PMID: 25684545; PubMed Central PMCID: PMCPMC4534335.
5. Jombart T, Devillard S, Balloux F. Discriminant analysis of principal components: A new method for the analysis of genetically structured populations. BMC Genet. 2010;11(1):94. PubMed PMID: doi:10.1186/1471-2156-11-94.
6. Jombart T. Adegenet: A R package for the multivariate analysis of genetic markers. Bioinformatics. 2008;24(11):1403-5. doi: 10.1093/bioinformatics/btn129.
7. R Core Team. R: A language and environment for statistical computing Vienna, Austria.: R Foundation for Statistical Computing; 2017. Available from: http://www.R-project.org/.
8. RStudio Team. RStudio: Integrated Development for R. Boston, MA: RStudio, Inc.; 2019.
9. Rodriguez-Ramilo ST, Wang J. The effect of close relatives on unsupervised Bayesian clustering algorithms in population genetic structure analysis. Mol Ecol Resour. 2012;12(5):873-84. doi: 10.1111/j.1755-0998.2012.03156.x.

10. de Jager D, Swarts P, Harper C, Bloomer P. Friends and family: A software program for identification of unrelated individuals from molecular marker data. Mol Ecol Resour. 2017;17(6):e225-e33. doi: 10.1111/1755-0998.12691. PubMed PMID: 28503747.

11. Rousset F. genepop'007: A complete re-implementation of the genepop software for Windows and Linux. Mol Ecol Resour. 2008;8(1):103-6. doi: 10.1111/j.1471-8286.2007.01931.x.

12. Rice WR. Analyzing tables of statistical tests. Evolution. 1989;43(1):223-5. doi: 10.2307/2409177.
