# Supplementary material for: Genetic diversity, relatedness and inbreeding of ranched and fragmented Cape buffalo populations in southern Africa
Source: PLoS One. 2020 Aug 14;15(8):e0236717. doi: 10.1371/journal.pone.0236717 (PMC7428177; doi:10.1371/journal.pone.0236717)
Supplement: S1 Fig — Vertical lines indicate 95% confidence intervals. Numbers inserted for GNP and GNP-MNP indicate the value of the upper bound of the 95% CI. The dashed line indicates the lower 95% CI of GNP. Values are also shown in S3 Table. PVT: Private ranches combined. (PDF) [file pone.0236717.s002.pdf]

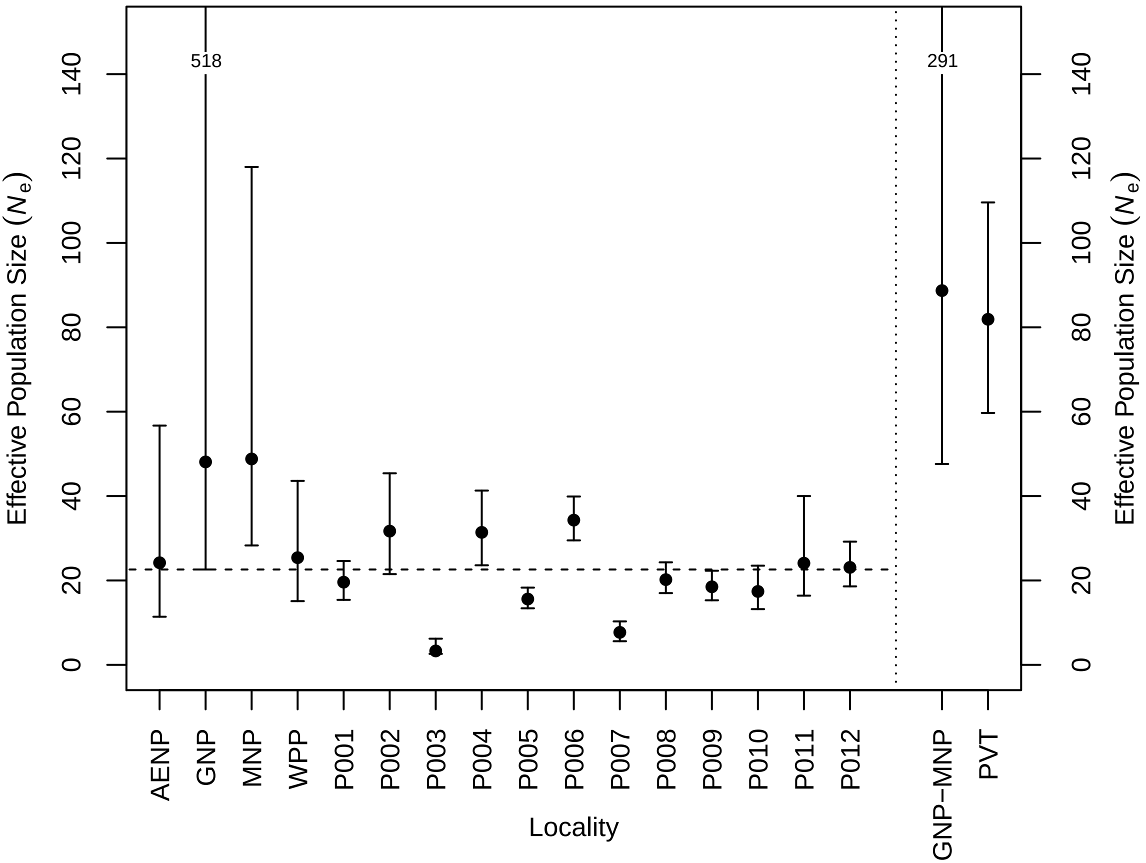

Effective Population Size (N_e)
0
20
40
60
80
100
120
140
518
291
AENP
GNP
MNP
WPP
P001
P002
P003
P004
P005
P006
P007
P008
P009
P010
P011
P012
GNP–MNP
PVT
Locality

S1 Fig. Estimated effective population size (N_e) of the buffalo population from each locality. Vertical lines indicate 95% confidence intervals. Numbers inserted for GNP and GNP-MNP indicate the value of the upper bound of the 95% CI. The dashed line indicates the lower 95% CI of GNP. Values are also shown in S3 Table. PVT: Private ranches combined.
