# Supplementary material for: Genetic diversity, relatedness and inbreeding of ranched and fragmented Cape buffalo populations in southern Africa
Source: PLoS One. 2020 Aug 14;15(8):e0236717. doi: 10.1371/journal.pone.0236717 (PMC7428177; doi:10.1371/journal.pone.0236717)
Supplement: S2 Fig — The first column of graphs [L(K)] show the mean log likelihood of each value of K with its associated standard deviation, while the second column (DeltaK) shows the most likely value of K as determined by the Evanno method. Rows indicate the full data set (FDS) and the relatives removed (RR) data set. The graphs were generated using StructureHarvester and further organized in Inkscape v0.92 (https://inkscape.org/). (PDF) [file pone.0236717.s003.pdf]

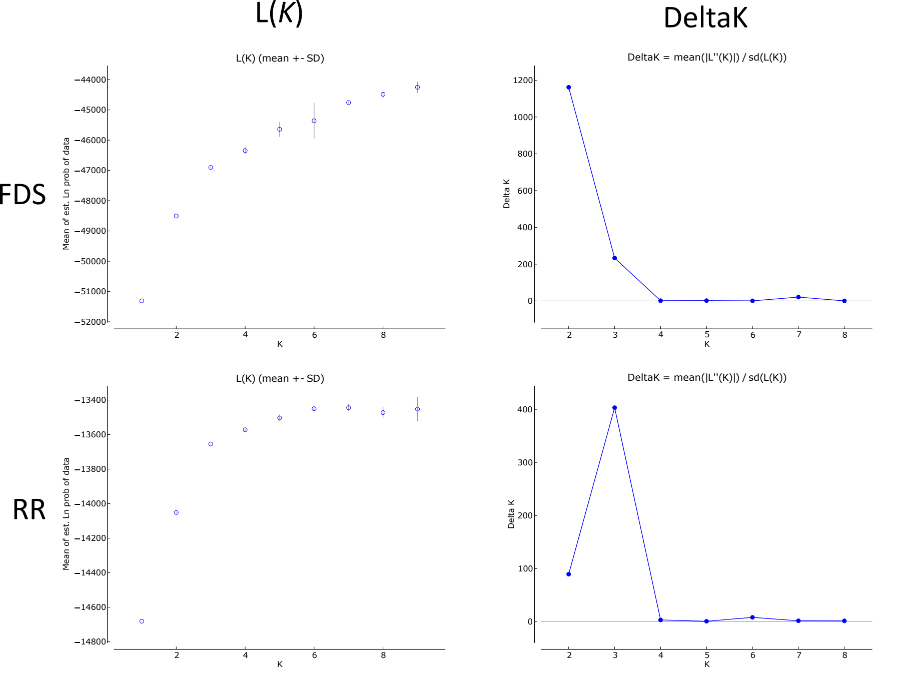

L(K)
DeltaK
FDS
RR
L(K) (mean +- SD)
Mean of est. Ln prob of data
DeltaK = mean(|L''(K)|) / sd(L(K))
Delta K
K

S2 Fig. Statistical support for *K*. The first column of graphs [L(*K*)] show the mean log likelihood of each value of *K* with its associated standard deviation, while the second column (DeltaK) shows the most likely value of *K* as determined by the Evanno method. Rows indicate the full data set (FDS) and the relatives removed (RR) data set. The graphs were generated using StructureHarvester and further organized in Inkscape v0.92 (https://inkscape.org/).
