# Supplementary material for: Genetic diversity, relatedness and inbreeding of ranched and fragmented Cape buffalo populations in southern Africa
Source: PLoS One. 2020 Aug 14;15(8):e0236717. doi: 10.1371/journal.pone.0236717 (PMC7428177; doi:10.1371/journal.pone.0236717)
Supplement: S3 Fig — A–full data set, B–relatives removed. The plots were generated using the online version of Clumpak and further organized in Inkscape v0.92 (https://inkscape.org/). (PDF) [file pone.0236717.s004.pdf]

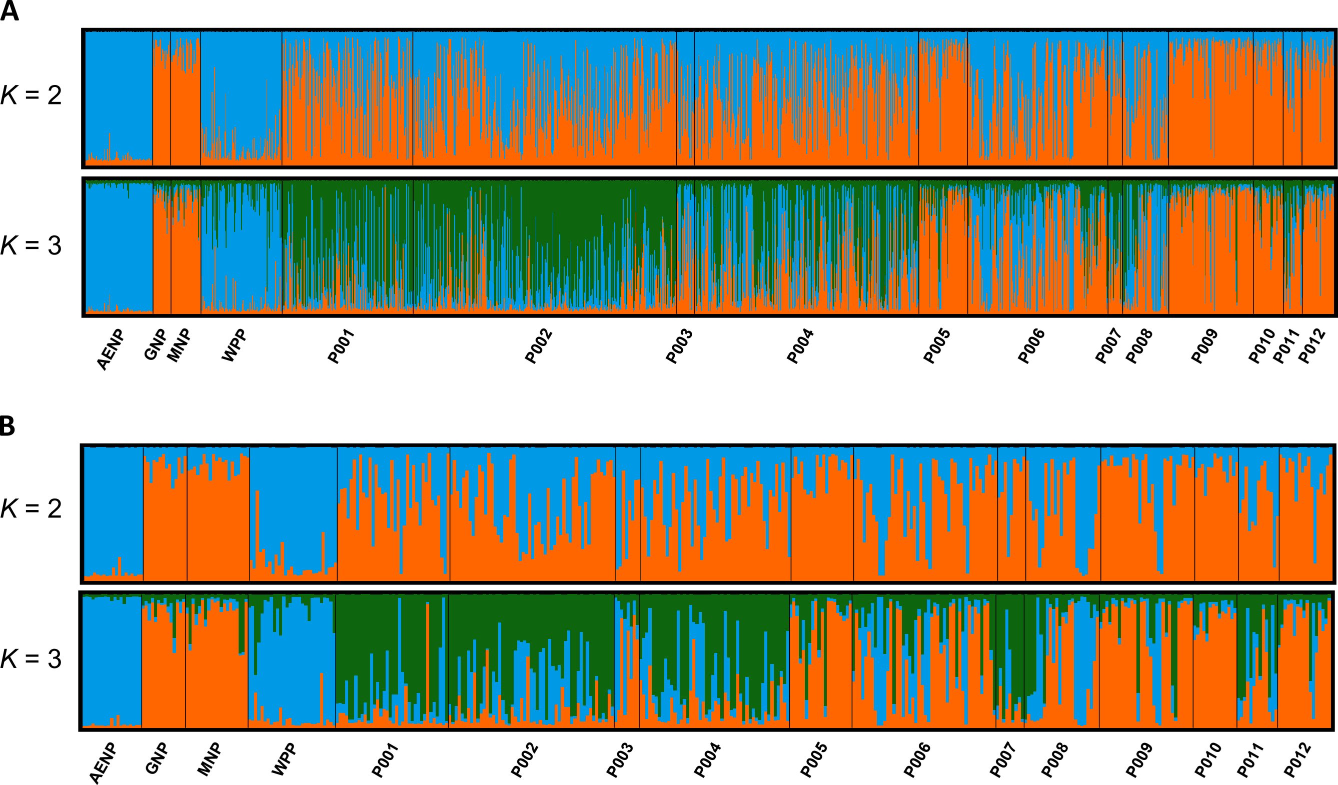
A
K = 2
K = 3
AENP
GNP
MNP
WPP
P001
P002
P003
P004
P005
P006
P007
P008
P009
P010
P011
P012
B
K = 2
K = 3
AENP
GNP
MNP
WPP
P001
P002
P003
P004
P005
P006
P007
P008
P009
P010
P011
P012

S3 Fig. Individual assignment plots of the STRUCTURE analyses at *K* = 2 and *K* = 3. A – full data set, **B** – relatives removed. The plots were generated using the online version of Clumpak and further organized in Inkscape v0.92 (https://inkscape.org/).
