# Supplementary material for: Genetic diversity, relatedness and inbreeding of ranched and fragmented Cape buffalo populations in southern Africa
Source: PLoS One. 2020 Aug 14;15(8):e0236717. doi: 10.1371/journal.pone.0236717 (PMC7428177; doi:10.1371/journal.pone.0236717)
Supplement: S4 Fig — AENP Cluster: Addo Elephant National Park cluster, GNP-MNP Cluster: Graspan and Mokala National Park cluster, “Other” Cluster: Third, unknown origin cluster. (PDF) [file pone.0236717.s005.pdf]

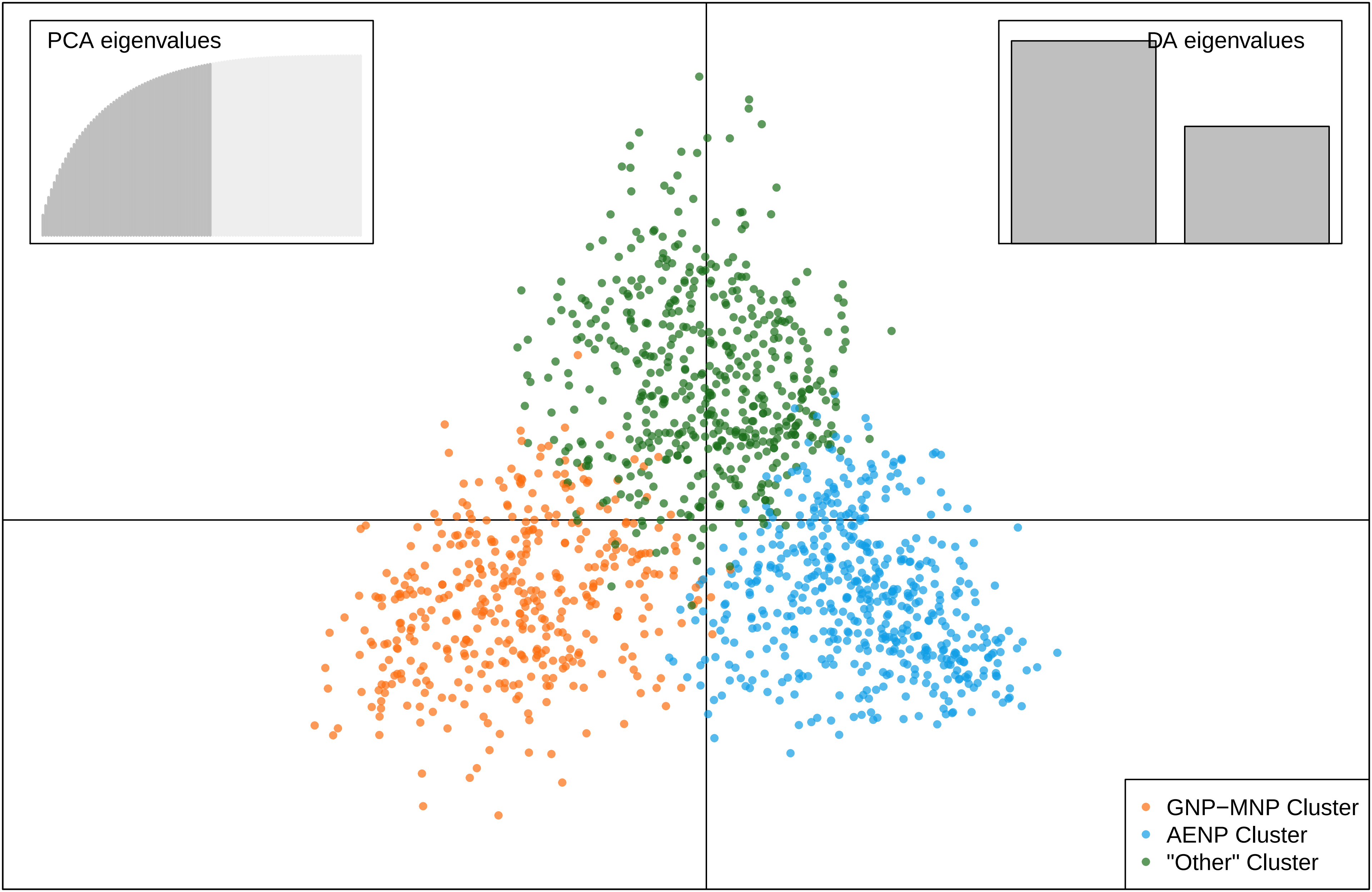
PCA eigenvalues
DA eigenvalues
GNP−MNP Cluster
AENP Cluster
"Other" Cluster

S4 Fig. Discriminant analysis of principal components (DAPC) of the full data set at *K* = 3. AENP Cluster: Addo Elephant National Park cluster, GNP-MNP Cluster: Graspan and Mokala National Park cluster, “Other” Cluster: Third, unknown origin cluster.
