# Supplementary material for: Genetic diversity, relatedness and inbreeding of ranched and fragmented Cape buffalo populations in southern Africa
Source: PLoS One. 2020 Aug 14;15(8):e0236717. doi: 10.1371/journal.pone.0236717 (PMC7428177; doi:10.1371/journal.pone.0236717)
Supplement: S1 Table — Calculated in Cervus v3.0.7. (DOCX) [file pone.0236717.s006.docx]

**S1 Table.** **Summary statistics of microsatellite loci used in this study.** Calculated in Cervus v3.0.7.

| Locus | *N*_A_ | *H*_O_ | *H*_E_ | PIC | Reference |
| --- | --- | --- | --- | --- | --- |
| BM1824 | 15 | 0.81 | 0.861 | 0.846 | van Hooft et al. [1] |
| BM3205 | 15 | 0.818 | 0.856 | 0.84 | van Hooft et al. [1] |
| BM3517 | 7 | 0.583 | 0.625 | 0.592 | van Hooft et al. [1] |
| BM719 | 15 | 0.83 | 0.862 | 0.849 | van Hooft et al. [1] |
| CSSM19 | 14 | 0.685 | 0.734 | 0.711 | Moore et al. [2] |
| ETH10 | 5 | 0.307 | 0.306 | 0.27 | Luikart et al. [3] |
| ILSTS026 | 13 | 0.718 | 0.815 | 0.797 | Kemp et al. [4] |
| INRA006 | 10 | 0.722 | 0.782 | 0.748 | Vaiman et al. [5] |
| SPS115 | 17 | 0.799 | 0.834 | 0.813 | Mommens et al. [6] |
| TGLA227 | 4 | 0.324 | 0.438 | 0.407 | Greyling et al. [7] |
| TGLA263 | 9 | 0.589 | 0.66 | 0.604 | Mommens et al. [6] |
| Amelogenin (Sex marker) | X, Y | - | - | - | Grzybowski et al. [8] |

*N*_A_: Number of alleles. *H*_O_: Observed heterozygosity. *H*_E_: Expected heterozygosity. PIC: Polymorphic information content.

**References**

1. van Hooft WF, Hanotte O, Wenink PW, Groen AF, Sugimoto Y, Prins HHT, et al. Applicability of bovine microsatellite markers for population genetic studies on African buffalo (*Syncerus caffer*). Anim Genet. 1999;30:214-20.

2. Moore SS, K. Byrne, Berger KT, Barendse W, McCarthy F, Womack JE, et al. Characterization of 65 bovine microsatellites. Mamm Genome. 1994;5:84-90.

3. Luikart G, Biju-Duval M-P, Ertugrul O, Zagdsuren Y, Maudet C, Taberlet P. Power of 22 microsatellite markers in fluorescent multiplexes for parentage testing in goats (*Capra hircus*). Anim Genet. 1999;30:431-8.

4. Kemp SJ, Hishida O, Wambugu J, Rink A, Longeri ML, Ma RZ, et al. A panel of polymorphic bovine, ovine and caprine microsatellite markers. Anim Genet. 1995;26:299-306.

5. Vaiman D, Osta R, Mercier D, Grohs C, Leveziel H. Characterization of five new bovine dinucleotide repeats. Anim Genet. 1992;23(6):537-41. doi: 10.1111/j.1365-2052.1992.tb00175.x.

6. Mommens G, Van Zeveren A, Peelman LJ. Effectiveness of bovine microsatellites in resolving paternity cases in American bison, *Bison bison* L. Anim Genet. 1998;29:12-8.

7. Greyling BJ, Kryger P, du Plessis S, van Hooft WF, van Helden P, Getz WM, et al. Development of a high-throughput microsatellite typing approach for forensic and population genetic analysis of wild and domestic African Bovini. African Journal of Biotechnology. 2008;7:655-60.

8. Grzybowski G, Prusak B, Romaniuk B. A novel variant of the amelogenin gene (*AMEL-X*) in cattle and its implications for sex determination. Anim Sci Pap Rep. 2006;24(2):111-8.
