# Supplementary material for: Genetic diversity, relatedness and inbreeding of ranched and fragmented Cape buffalo populations in southern Africa
Source: PLoS One. 2020 Aug 14;15(8):e0236717. doi: 10.1371/journal.pone.0236717 (PMC7428177; doi:10.1371/journal.pone.0236717)
Supplement: S2 Table — TrioML (values in bold) had the lowest variance for each sampling locality and produces positive relatedness estimates between zero and one (as does DyadML). (DOCX) [file pone.0236717.s007.docx]

**S2 Table.** **Mean and variance of the relatedness estimators available in COANCESTRY.** TrioML (values in bold) had the lowest variance for each sampling locality and produces positive relatedness estimates between zero and one (as does DyadML).

| **Locality** | **-** | **TrioML** | **Wang** | **LynchLi** | **LynchRd** | **Ritland** | **QuellerGt** | **DyadML** |
| --- | --- | --- | --- | --- | --- | --- | --- | --- |
| **AENP** | Mean | **0.096** | -0.041 | -0.038 | -0.015 | -0.014 | -0.012 | 0.120 |
|  | Variance | **0.023** | 0.134 | 0.132 | 0.060 | 0.065 | 0.093 | 0.031 |
| **GNP** | Mean | **0.044** | -0.005 | -0.012 | -0.051 | -0.050 | -0.051 | 0.054 |
|  | Variance | **0.009** | 0.039 | 0.047 | 0.020 | 0.022 | 0.043 | 0.012 |
| **MNP** | Mean | **0.049** | -0.019 | -0.017 | -0.030 | -0.031 | -0.023 | 0.063 |
|  | Variance | **0.008** | 0.045 | 0.054 | 0.019 | 0.024 | 0.041 | 0.012 |
| **WPP** | Mean | **0.070** | -0.019 | -0.015 | -0.011 | -0.011 | -0.009 | 0.089 |
|  | Variance | **0.014** | 0.072 | 0.081 | 0.031 | 0.034 | 0.063 | 0.020 |
| **P001** | Mean | **0.072** | -0.005 | -0.001 | -0.007 | -0.007 | -0.008 | 0.085 |
|  | Variance | **0.016** | 0.051 | 0.053 | 0.030 | 0.027 | 0.046 | 0.020 |
| **P002** | Mean | **0.071** | 0.001 | -0.003 | -0.003 | -0.003 | -0.002 | 0.085 |
|  | Variance | **0.015** | 0.054 | 0.055 | 0.027 | 0.024 | 0.045 | 0.019 |
| **P003** | Mean | **0.115** | -0.019 | -0.019 | -0.050 | -0.052 | -0.032 | 0.131 |
|  | Variance | **0.031** | 0.152 | 0.154 | 0.071 | 0.056 | 0.103 | 0.039 |
| **P004** | Mean | **0.078** | -0.013 | -0.011 | -0.004 | -0.004 | -0.002 | 0.093 |
|  | Variance | **0.018** | 0.070 | 0.074 | 0.030 | 0.021 | 0.056 | 0.023 |
| **P005** | Mean | **0.069** | 0.008 | -0.002 | -0.018 | -0.018 | -0.014 | 0.083 |
|  | Variance | **0.019** | 0.055 | 0.060 | 0.030 | 0.025 | 0.053 | 0.024 |
| **P006** | Mean | **0.062** | -0.031 | -0.027 | -0.007 | -0.007 | -0.008 | 0.076 |
|  | Variance | **0.013** | 0.052 | 0.060 | 0.024 | 0.026 | 0.048 | 0.016 |
| **P007** | Mean | **0.065** | 0.029 | 0.018 | -0.063 | -0.058 | -0.058 | 0.081 |
|  | Variance | **0.020** | 0.075 | 0.074 | 0.040 | 0.034 | 0.076 | 0.026 |
| **P008** | Mean | **0.067** | -0.016 | -0.021 | -0.019 | -0.019 | -0.019 | 0.082 |
|  | Variance | **0.016** | 0.060 | 0.070 | 0.029 | 0.023 | 0.061 | 0.021 |
| **P009** | Mean | **0.077** | 0.003 | -0.006 | -0.010 | -0.011 | -0.004 | 0.092 |
|  | Variance | **0.019** | 0.070 | 0.073 | 0.033 | 0.031 | 0.054 | 0.024 |
| **P010** | Mean | **0.075** | -0.001 | -0.002 | -0.031 | -0.032 | -0.029 | 0.089 |
|  | Variance | **0.020** | 0.056 | 0.054 | 0.035 | 0.039 | 0.051 | 0.025 |
| **P011** | Mean | **0.059** | 0.016 | 0.003 | -0.050 | -0.050 | -0.051 | 0.072 |
|  | Variance | **0.014** | 0.055 | 0.054 | 0.027 | 0.024 | 0.046 | 0.019 |
| **P012** | Mean | **0.067** | 0.002 | 0.007 | -0.028 | -0.027 | -0.024 | 0.080 |
|  | Variance | **0.018** | 0.058 | 0.061 | 0.027 | 0.022 | 0.050 | 0.022 |
