# Supplementary material for: Genetic diversity, relatedness and inbreeding of ranched and fragmented Cape buffalo populations in southern Africa
Source: PLoS One. 2020 Aug 14;15(8):e0236717. doi: 10.1371/journal.pone.0236717 (PMC7428177; doi:10.1371/journal.pone.0236717)
Supplement: S3 Table — (DOCX) [file pone.0236717.s008.docx]

**S3 Table.** **Population summary statistics for each sampling locality.**

| **Locality** | ***N*** | ***H*_O_ (SD)** | ***H*_E_ (SD)** | ***A*_R_ (95% CI)** | ***F*_IS_ (95% CI)** | ***N*_e_ (95% CI)** | **Pooled *N*_e_ (95% CI)** |
| --- | --- | --- | --- | --- | --- | --- | --- |
| **AENP** | 79 | 0.402 (0.239) | 0.425 (0.24) | 3.07 (2.8182 - 3.2727) | 0.0489 (-0.005 - 0.1079) | 24.2 (11.4 - 56.7) |  |
| **GNP** | 21 | 0.658 (0.227) | 0.676 (0.194) | 5.37 (4.7273 - 5.8182) | 0.0017 (-0.0802 - 0.0831) | 48.1 (22.6 - 518.4) | 88.7 (47.6 - 290.5) |
| **MNP** | 35 | 0.614 (0.231) | 0.659 (0.23) | 5.83 (5.1818 - 6.3659) | 0.0545 (-0.0096 - 0.1186) | 48.8 (28.3 - 118.0) |  |
| **WPP** | 95 | 0.55 (0.194) | 0.563 (0.192) | 4.63 (4 - 5.1818) | 0.0184 (-0.0205 - 0.0561) | 25.4 (15.1 - 43.6) |  |
| **P001** | 153 | 0.724 (0.166) | 0.729 (0.169) | 6.42 (5.7273 - 7) | 0.0036 (-0.027 - 0.0357) | 19.6 (15.4 - 24.6) | 81.9 (59.7 - 109.6) |
| **P002** | 308 | 0.692 (0.217) | 0.689 (0.204) | 6.24 (5.5455 - 6.9091) | -0.0063 (-0.0268 - 0.0137) | 31.7 (21.5 - 45.4) |  |
| **P003** | 21 | 0.638 (0.193) | 0.67 (0.165) | 4.59 (3.9091 - 5.1818) | 0.0151 (-0.1393 - 0.15) | 3.3 (2.6 - 6.2) |  |
| **P004** | 262 | 0.677 (0.171) | 0.695 (0.157) | 6.03 (5.2727 - 6.7273) | 0.0239 (-0.0002 - 0.048) | 31.4 (23.6 - 41.3) |  |
| **P005** | 57 | 0.664 (0.262) | 0.658 (0.231) | 6.15 (5.3636 - 6.8182) | -0.0186 (-0.0591 - 0.0276) | 15.6 (13.4 - 18.3) |  |
| **P006** | 164 | 0.664 (0.165) | 0.708 (0.157) | 6.44 (5.6364 - 7.1818) | 0.0591 (0.0277 - 0.092) | 34.3 (29.5 - 39.9) |  |
| **P007** | 17 | 0.722 (0.206) | 0.687 (0.178) | 5.33 (4.7273 - 5.7273) | -0.0823 (-0.1699 - -0.0046) | 7.7 (5.6 - 10.3) |  |
| **P008** | 54 | 0.638 (0.225) | 0.665 (0.189) | 5.65 (5 - 6.2727) | 0.0316 (-0.0142 - 0.0789) | 20.2 (17 - 24.3) |  |
| **P009** | 99 | 0.633 (0.267) | 0.638 (0.243) | 5.75 (4.8182 - 6.5455) | 0.0025 (-0.0386 - 0.0427) | 18.5 (15.3 - 22.3) |  |
| **P010** | 35 | 0.668 (0.273) | 0.67 (0.244) | 5.48 (5 - 6) | -0.0122 (-0.0666 - 0.0394) | 17.4 (13.2 - 23.5) |  |
| **P011** | 22 | 0.689 (0.231) | 0.693 (0.187) | 5.38 (4.6364 - 6) | -0.0212 (-0.1012 - 0.047) | 24.1 (16.4 - 40.0) |  |
| **P012** | 37 | 0.651 (0.233) | 0.641 (0.244) | 5.7 (5.0886 - 6.2727) | -0.0286 (-0.0847 - 0.0257) | 23.1 (18.6 - 29.2) |  |

*N*: Sample size, *H*_O_ (SD): Observed heterozygosity (Standard deviation), *H*_E_ (SD): Expected heterozygosity (Standard deviation), *A*_R_ (95% CI): Allelic richness (95% confidence interval), *F*_IS_ (95% CI): Inbreeding coefficient (95% confidence interval), *N*_E_ (95% CI): Effective population size (95% confidence interval), Combined *N*_E_ (95% CI): Effective population size of certain localities combined (95% confidence interval)
