# Supplementary material for: Genetic diversity, relatedness and inbreeding of ranched and fragmented Cape buffalo populations in southern Africa
Source: PLoS One. 2020 Aug 14;15(8):e0236717. doi: 10.1371/journal.pone.0236717 (PMC7428177; doi:10.1371/journal.pone.0236717)
Supplement: S4 Table — (DOCX) [file pone.0236717.s009.docx]

**S4 Table. Relatedness and individual inbreeding statistics.**

| **Locality** | **Mean *r*** | **Variance *r*** | **Skewness *r*** | **Kurtosis *r*** | **Proportion *r* >= 0.25** | **Mean *F*** | **Variance *F*** |
| --- | --- | --- | --- | --- | --- | --- | --- |
| **AENP** | 0.096 | 0.023 | 1.75 | 5.14 | 0.145 | 0.156 | 0.035 |
| **GNP** | 0.044 | 0.009 | 3.31 | 14.64 | 0.048 | 0.080 | 0.014 |
| **MNP** | 0.049 | 0.008 | 2.81 | 12.56 | 0.042 | 0.097 | 0.016 |
| **WPP** | 0.070 | 0.014 | 2.22 | 7.70 | 0.087 | 0.089 | 0.014 |
| **P001** | 0.072 | 0.016 | 2.25 | 7.89 | 0.099 | 0.086 | 0.018 |
| **P002** | 0.071 | 0.015 | 2.25 | 7.98 | 0.094 | 0.068 | 0.011 |
| **P003** | 0.115 | 0.032 | 1.36 | 3.49 | 0.205 | 0.083 | 0.019 |
| **P004** | 0.078 | 0.018 | 2.17 | 7.37 | 0.108 | 0.095 | 0.017 |
| **P005** | 0.069 | 0.019 | 2.63 | 10.26 | 0.098 | 0.055 | 0.009 |
| **P006** | 0.062 | 0.013 | 2.52 | 9.72 | 0.072 | 0.112 | 0.024 |
| **P007** | 0.065 | 0.021 | 2.46 | 8.20 | 0.088 | 0.023 | 0.002 |
| **P008** | 0.067 | 0.016 | 2.45 | 8.77 | 0.084 | 0.097 | 0.020 |
| **P009** | 0.077 | 0.019 | 2.19 | 7.24 | 0.110 | 0.073 | 0.013 |
| **P010** | 0.075 | 0.020 | 2.14 | 6.55 | 0.118 | 0.080 | 0.008 |
| **P011** | 0.059 | 0.014 | 2.57 | 9.37 | 0.082 | 0.072 | 0.007 |
| **P012** | 0.067 | 0.018 | 2.33 | 7.44 | 0.101 | 0.047 | 0.006 |

*r*: TrioML relatedness, *F*: TrioML inbreeding coefficient.
