# Supplementary material for: Genetic diversity, relatedness and inbreeding of ranched and fragmented Cape buffalo populations in southern Africa
Source: PLoS One. 2020 Aug 14;15(8):e0236717. doi: 10.1371/journal.pone.0236717 (PMC7428177; doi:10.1371/journal.pone.0236717)
Supplement: S5 Table — (DOCX) [file pone.0236717.s010.docx]

**S5 Table**. **Mean relatedness within sexes.**

| **Locality** | ***N*** | ***N*_females_** | ***N*_males_** | ***N*_females_:*N*_males_** | **Mean *r*_females_** | **Mean *r*_males_** |
| --- | --- | --- | --- | --- | --- | --- |
| **AENP** | 79 | 56 | 23 | 2.43 | 0.094 | 0.105 |
| **GNP** | 21 | 9 | 12 | 0.75 | 0.032 | 0.038 |
| **MNP** | 35 | 16 | 19 | 0.84 | 0.055 | 0.058 |
| **WPP** | 95 | 52 | 43 | 1.21 | 0.070 | 0.084 |
| **P001** | 152 | 85 | 67 | 1.27 | 0.071 | 0.086 |
| **P002** | 306 | 186 | 120 | 1.55 | 0.071 | 0.077 |
| **P003** | 21 | 9 | 12 | 0.75 | 0.158 | 0.095 |
| **P004** | 261 | 142 | 119 | 1.19 | 0.080 | 0.082 |
| **P005** | 57 | 37 | 20 | 1.85 | 0.068 | 0.080 |
| **P006** | 164 | 109 | 55 | 1.98 | 0.065 | 0.072 |
| **P007** | 17 | 15 | 2 | 7.50 | 0.071 | 0.240 |
| **P008** | 54 | 41 | 13 | 3.15 | 0.062 | 0.114 |
| **P009** | 99 | 60 | 39 | 1.54 | 0.067 | 0.102 |
| **P010** | 35 | 34 | 1 | 34.0 | 0.075 | NA |
| **P011** | 22 | 14 | 8 | 1.75 | 0.083 | 0.046 |
| **P012** | 37 | 27 | 10 | 2.70 | 0.056 | 0.116 |
| **Total/*Mean*** | 1455* | 892 | 563 | *1.58* | *0.074* | *0.093* |

*N*: Sample size, *N*_females_: female sample size, *N*_males_: male sample size, *r*: relatedness coefficient (TrioML) NA: Unable to calculate pairwise relatedness within males, as only one male was present in the data set. *Four samples were excluded from these analyses. Three samples had missing data at the sex marker and one was genotyped as YY and thus excluded.
