# Supplementary material for: Genetic diversity, relatedness and inbreeding of ranched and fragmented Cape buffalo populations in southern Africa
Source: PLoS One. 2020 Aug 14;15(8):e0236717. doi: 10.1371/journal.pone.0236717 (PMC7428177; doi:10.1371/journal.pone.0236717)
Supplement: S6 Table — (DOCX) [file pone.0236717.s011.docx]

**S6 Table.** **Pairwise *D*_JOST_ and *F*_ST_ values with 95% confidence intervals.**

|  | *D*_JOST_ | | | *F*_ST_ | | |
| --- | --- | --- | --- | --- | --- | --- |
| Locality pair | Actual estimate | Lower 95% CI | Upper 95% CI | Actual estimate | Lower 95% CI | Upper 95% CI |
| AENP vs GNP | 0.2893 | 0.2423 | 0.3345 | 0.2549 | 0.2245 | 0.2838 |
| AENP vs MNP | 0.3032 | 0.2608 | 0.3504 | 0.2566 | 0.2326 | 0.2827 |
| AENP vs WPP | 0.0382 | 0.0231 | 0.0558 | 0.0529 | 0.0372 | 0.0698 |
| AENP vs P001 | 0.2215 | 0.1891 | 0.2549 | 0.1436 | 0.1264 | 0.1613 |
| AENP vs P002 | 0.1664 | 0.1448 | 0.1893 | 0.1351 | 0.1221 | 0.1474 |
| AENP vs P003 | 0.0663 | -0.0102 | 0.1438 | 0.1286 | 0.0865 | 0.1753 |
| AENP vs P004 | 0.1306 | 0.1091 | 0.153 | 0.1115 | 0.0988 | 0.1252 |
| AENP vs P005 | 0.2873 | 0.2486 | 0.3256 | 0.2478 | 0.2191 | 0.278 |
| AENP vs P006 | 0.1371 | 0.1099 | 0.1667 | 0.1064 | 0.0902 | 0.1226 |
| AENP vs P007 | 0.2214 | 0.1423 | 0.3288 | 0.2292 | 0.1814 | 0.2831 |
| AENP vs P008 | 0.0948 | 0.0616 | 0.1326 | 0.0991 | 0.0735 | 0.1289 |
| AENP vs P009 | 0.3069 | 0.2747 | 0.34 | 0.2472 | 0.2246 | 0.2691 |
| AENP vs P010 | 0.31 | 0.2632 | 0.3592 | 0.2717 | 0.2469 | 0.3005 |
| AENP vs P011 | 0.207 | 0.1428 | 0.2814 | 0.1983 | 0.158 | 0.2431 |
| AENP vs P012 | 0.2858 | 0.2314 | 0.3379 | 0.2652 | 0.2321 | 0.2973 |
| GNP vs MNP | 0.0026 | -0.022 | 0.0415 | 0.0049 | -0.0128 | 0.0292 |
| GNP vs WPP | 0.2269 | 0.1846 | 0.2738 | 0.1562 | 0.1328 | 0.1803 |
| GNP vs P001 | 0.1122 | 0.0788 | 0.1551 | 0.0603 | 0.0467 | 0.0772 |
| GNP vs P002 | 0.1362 | 0.1018 | 0.1753 | 0.0719 | 0.057 | 0.0894 |
| GNP vs P003 | 0.0774 | -0.0062 | 0.1619 | 0.0687 | 0.0262 | 0.1206 |
| GNP vs P004 | 0.143 | 0.107 | 0.1794 | 0.0698 | 0.0546 | 0.0876 |
| GNP vs P005 | 0.0583 | 0.0248 | 0.1022 | 0.0379 | 0.0171 | 0.0647 |
| GNP vs P006 | 0.0636 | 0.0342 | 0.101 | 0.0409 | 0.0261 | 0.0588 |
| GNP vs P007 | 0.1131 | 0.061 | 0.1874 | 0.0757 | 0.0525 | 0.1074 |
| GNP vs P008 | 0.0903 | 0.0476 | 0.1402 | 0.0587 | 0.0341 | 0.0861 |
| GNP vs P009 | 0.0187 | -0.001 | 0.0481 | 0.0247 | 0.01 | 0.0439 |
| GNP vs P010 | 0.0452 | 0.0089 | 0.0943 | 0.0258 | 0.0063 | 0.0523 |
| GNP vs P011 | 0.0523 | 0.0116 | 0.1041 | 0.0289 | 0.0096 | 0.0543 |
| GNP vs P012 | 0.048 | 0.0094 | 0.0983 | 0.0366 | 0.0134 | 0.0651 |
| MNP vs WPP | 0.2456 | 0.2046 | 0.29 | 0.1548 | 0.1341 | 0.1761 |
| MNP vs P001 | 0.1414 | 0.1089 | 0.18 | 0.0743 | 0.0607 | 0.089 |
| MNP vs P002 | 0.1725 | 0.1389 | 0.2091 | 0.0822 | 0.0686 | 0.0989 |
| MNP vs P003 | 0.1251 | 0.0461 | 0.2124 | 0.075 | 0.0356 | 0.1234 |
| MNP vs P004 | 0.147 | 0.1205 | 0.1753 | 0.0778 | 0.065 | 0.0921 |
| MNP vs P005 | 0.0554 | 0.0249 | 0.0919 | 0.0384 | 0.02 | 0.0615 |
| MNP vs P006 | 0.0809 | 0.049 | 0.1185 | 0.0492 | 0.035 | 0.0646 |
| MNP vs P007 | 0.0862 | 0.0478 | 0.14 | 0.0799 | 0.0589 | 0.1055 |
| MNP vs P008 | 0.1112 | 0.0655 | 0.1644 | 0.0622 | 0.0386 | 0.0889 |
| MNP vs P009 | 0.0251 | 0.0066 | 0.0508 | 0.0213 | 0.01 | 0.0365 |
| MNP vs P010 | 0.0282 | -0.0035 | 0.0674 | 0.0225 | 0.0068 | 0.0445 |
| MNP vs P011 | 0.0787 | 0.0406 | 0.122 | 0.0371 | 0.0182 | 0.0597 |
| MNP vs P012 | 0.0202 | -0.0046 | 0.0547 | 0.0184 | 0.0022 | 0.0388 |
| WPP vs P001 | 0.1381 | 0.1112 | 0.1682 | 0.0786 | 0.0641 | 0.0939 |
| WPP vs P002 | 0.1099 | 0.0915 | 0.1296 | 0.074 | 0.0635 | 0.0849 |
| WPP vs P003 | 0.0494 | -0.0096 | 0.1119 | 0.0694 | 0.0399 | 0.1056 |
| WPP vs P004 | 0.1243 | 0.1014 | 0.1472 | 0.0748 | 0.063 | 0.0869 |
| WPP vs P005 | 0.2331 | 0.1915 | 0.2757 | 0.1594 | 0.1321 | 0.1894 |
| WPP vs P006 | 0.0928 | 0.0712 | 0.1151 | 0.0554 | 0.0434 | 0.0679 |
| WPP vs P007 | 0.1916 | 0.1325 | 0.2706 | 0.1308 | 0.1012 | 0.1695 |
| WPP vs P008 | 0.0808 | 0.0511 | 0.1161 | 0.0518 | 0.035 | 0.0709 |
| WPP vs P009 | 0.274 | 0.2411 | 0.3053 | 0.1691 | 0.1482 | 0.1895 |
| WPP vs P010 | 0.2436 | 0.202 | 0.2891 | 0.1652 | 0.1433 | 0.1882 |
| WPP vs P011 | 0.1414 | 0.0849 | 0.2109 | 0.1106 | 0.0761 | 0.1472 |
| WPP vs P012 | 0.2546 | 0.2088 | 0.3005 | 0.1727 | 0.1447 | 0.2021 |
| P001 vs P002 | 0.0294 | 0.0177 | 0.0446 | 0.0163 | 0.0113 | 0.022 |
| P001 vs P003 | 0.0813 | 0.0008 | 0.1725 | 0.0536 | 0.0269 | 0.0903 |
| P001 vs P004 | 0.0535 | 0.0344 | 0.075 | 0.0246 | 0.0178 | 0.0323 |
| P001 vs P005 | 0.1455 | 0.1075 | 0.186 | 0.064 | 0.0486 | 0.0815 |
| P001 vs P006 | 0.068 | 0.0485 | 0.0899 | 0.0278 | 0.0212 | 0.0348 |
| P001 vs P007 | 0.0552 | 0.0231 | 0.0987 | 0.0278 | 0.0123 | 0.0482 |
| P001 vs P008 | 0.0862 | 0.0535 | 0.1228 | 0.0381 | 0.026 | 0.0528 |
| P001 vs P009 | 0.1667 | 0.1388 | 0.1963 | 0.078 | 0.0652 | 0.0921 |
| P001 vs P010 | 0.1384 | 0.1006 | 0.1898 | 0.0693 | 0.058 | 0.0834 |
| P001 vs P011 | 0.0469 | 0.0104 | 0.0941 | 0.0303 | 0.0156 | 0.0504 |
| P001 vs P012 | 0.1637 | 0.1237 | 0.2097 | 0.082 | 0.0655 | 0.101 |
| P002 vs P003 | 0.0611 | 0.0053 | 0.1315 | 0.0592 | 0.031 | 0.0976 |
| P002 vs P004 | 0.0566 | 0.0425 | 0.0717 | 0.0291 | 0.0232 | 0.0362 |
| P002 vs P005 | 0.1481 | 0.112 | 0.1846 | 0.0667 | 0.051 | 0.0844 |
| P002 vs P006 | 0.057 | 0.0447 | 0.0717 | 0.0336 | 0.0283 | 0.0397 |
| P002 vs P007 | 0.0706 | 0.0301 | 0.1213 | 0.0406 | 0.0233 | 0.0626 |
| P002 vs P008 | 0.0661 | 0.0438 | 0.0915 | 0.0354 | 0.0243 | 0.0486 |
| P002 vs P009 | 0.1751 | 0.1498 | 0.2009 | 0.0828 | 0.0705 | 0.0964 |
| P002 vs P010 | 0.1542 | 0.123 | 0.1926 | 0.0771 | 0.0659 | 0.0898 |
| P002 vs P011 | 0.0739 | 0.0354 | 0.1252 | 0.0429 | 0.0281 | 0.0631 |
| P002 vs P012 | 0.1782 | 0.143 | 0.2171 | 0.0901 | 0.0735 | 0.1093 |
| P003 vs P004 | 0.0758 | -0.0003 | 0.1354 | 0.0497 | 0.0268 | 0.0827 |
| P003 vs P005 | 0.1239 | 0.0361 | 0.2073 | 0.0851 | 0.0423 | 0.1331 |
| P003 vs P006 | 0.0119 | -0.0246 | 0.062 | 0.0138 | -0.0056 | 0.0444 |
| P003 vs P007 | 0.1291 | 0.0571 | 0.2166 | 0.0904 | 0.0484 | 0.1451 |
| P003 vs P008 | 0.0085 | -0.0379 | 0.0647 | 0.0224 | -0.0039 | 0.0603 |
| P003 vs P009 | 0.1302 | 0.0393 | 0.2109 | 0.0938 | 0.0504 | 0.1455 |
| P003 vs P010 | 0.1107 | 0.0081 | 0.2192 | 0.0895 | 0.0416 | 0.1473 |
| P003 vs P011 | 0.0786 | 0.0034 | 0.18 | 0.0415 | -0.0006 | 0.0966 |
| P003 vs P012 | 0.1671 | 0.0677 | 0.2723 | 0.1035 | 0.0597 | 0.157 |
| P004 vs P005 | 0.1343 | 0.0976 | 0.1732 | 0.0638 | 0.0494 | 0.0806 |
| P004 vs P006 | 0.0434 | 0.0317 | 0.0573 | 0.0247 | 0.0199 | 0.0303 |
| P004 vs P007 | 0.058 | 0.0255 | 0.1106 | 0.0468 | 0.0286 | 0.0714 |
| P004 vs P008 | 0.04 | 0.0264 | 0.057 | 0.0252 | 0.0176 | 0.0341 |
| P004 vs P009 | 0.154 | 0.129 | 0.1785 | 0.0763 | 0.0638 | 0.0897 |
| P004 vs P010 | 0.1728 | 0.1431 | 0.2071 | 0.0753 | 0.0626 | 0.0889 |
| P004 vs P011 | 0.0851 | 0.039 | 0.1374 | 0.0418 | 0.0227 | 0.0628 |
| P004 vs P012 | 0.1393 | 0.1059 | 0.1769 | 0.0706 | 0.0562 | 0.0871 |
| P005 vs P006 | 0.1243 | 0.0855 | 0.1682 | 0.0557 | 0.0383 | 0.0765 |
| P005 vs P007 | 0.1182 | 0.0657 | 0.18 | 0.0752 | 0.0485 | 0.1068 |
| P005 vs P008 | 0.1201 | 0.0751 | 0.1668 | 0.0648 | 0.0415 | 0.0913 |
| P005 vs P009 | 0.0668 | 0.0427 | 0.0947 | 0.0471 | 0.0338 | 0.0614 |
| P005 vs P010 | 0.0738 | 0.0408 | 0.1115 | 0.0457 | 0.0295 | 0.0634 |
| P005 vs P011 | 0.088 | 0.0562 | 0.1285 | 0.0501 | 0.0305 | 0.0745 |
| P005 vs P012 | 0.1039 | 0.0661 | 0.1456 | 0.057 | 0.0359 | 0.0808 |
| P006 vs P007 | 0.0719 | 0.0308 | 0.124 | 0.0447 | 0.0257 | 0.0727 |
| P006 vs P008 | 0.0231 | 0.0085 | 0.041 | 0.0141 | 0.0069 | 0.0228 |
| P006 vs P009 | 0.1193 | 0.0887 | 0.1517 | 0.0606 | 0.0457 | 0.0767 |
| P006 vs P010 | 0.1377 | 0.0973 | 0.1824 | 0.0606 | 0.0454 | 0.0772 |
| P006 vs P011 | 0.0444 | 0.0121 | 0.0872 | 0.0242 | 0.0098 | 0.0421 |
| P006 vs P012 | 0.1368 | 0.0975 | 0.1824 | 0.0657 | 0.0468 | 0.0878 |
| P007 vs P008 | 0.1107 | 0.0538 | 0.1786 | 0.0689 | 0.0405 | 0.1037 |
| P007 vs P009 | 0.1185 | 0.0877 | 0.1596 | 0.0763 | 0.0575 | 0.1015 |
| P007 vs P010 | 0.1837 | 0.1281 | 0.2435 | 0.0917 | 0.0693 | 0.1205 |
| P007 vs P011 | 0.0983 | 0.0391 | 0.1714 | 0.0623 | 0.0338 | 0.0944 |
| P007 vs P012 | 0.1367 | 0.0843 | 0.201 | 0.0937 | 0.0677 | 0.1262 |
| P008 vs P009 | 0.129 | 0.0811 | 0.1809 | 0.0726 | 0.0504 | 0.0984 |
| P008 vs P010 | 0.1212 | 0.0716 | 0.1801 | 0.0684 | 0.0419 | 0.0979 |
| P008 vs P011 | 0.0708 | 0.0246 | 0.1286 | 0.0409 | 0.0181 | 0.0684 |
| P008 vs P012 | 0.1564 | 0.1034 | 0.2122 | 0.0773 | 0.0493 | 0.1109 |
| P009 vs P010 | 0.0594 | 0.0335 | 0.0918 | 0.0312 | 0.0194 | 0.0464 |
| P009 vs P011 | 0.0953 | 0.0563 | 0.1367 | 0.0603 | 0.0401 | 0.0825 |
| P009 vs P012 | 0.0321 | 0.0138 | 0.0546 | 0.0318 | 0.0193 | 0.0475 |
| P010 vs P011 | 0.0855 | 0.0416 | 0.1403 | 0.043 | 0.0245 | 0.0659 |
| P010 vs P012 | 0.0341 | 0.0081 | 0.0725 | 0.027 | 0.0132 | 0.0464 |
| P011 vs P012 | 0.066 | 0.0257 | 0.1155 | 0.044 | 0.0204 | 0.0741 |
