# Supplementary material for: Genetic diversity, relatedness and inbreeding of ranched and fragmented Cape buffalo populations in southern Africa
Source: PLoS One. 2020 Aug 14;15(8):e0236717. doi: 10.1371/journal.pone.0236717 (PMC7428177; doi:10.1371/journal.pone.0236717)
Supplement: S7 Table — Data sets from sampling localities conformed to HWE after relatives were removed. (DOCX) [file pone.0236717.s012.docx]

**S7 Table. Hardy-Weinberg equilibrium (HWE) probability tests of each sampling locality, with the full data set and relatives removed.** Data sets from sampling localities conformed to HWE after relatives were removed.

| **Full data set** | | | | **Relatives removed** | | | |
| --- | --- | --- | --- | --- | --- | --- | --- |
| **Locality** | ***N*** | **HWE *p*-value** | **Bonferroni corrected *p*-value** | **Locality** | ***N*** | **HWE *p*-value** | **Bonferroni corrected *p*-value** |
| **AENP** | 79 | 0.0295* | 0.472 | **AENP** | 19 | 0.0576 | 0.9216 |
| **GNP** | 21 | 0.9252 | 1 | **GNP** | 14 | 0.7145 | 1 |
| **MNP** | 35 | 0.0042** | 0.0736 | **MNP** | 20 | 0.2237 | 1 |
| **WPP** | 95 | 0.0936 | 1 | **WPP** | 28 | 0.0149* | 0.2384 |
| **P001** | 153 | 0.0116* | 0.1776 | **P001** | 36 | 0.9034 | 1 |
| **P002** | 308 | 0*** | 0*** | **P002** | 53 | 0.6438 | 1 |
| **P003** | 21 | 0.0001*** | 0.0016** | **P003** | 8 | 0.9798 | 1 |
| **P004** | 262 | 0*** | 0*** | **P004** | 48 | 0.067 | 1 |
| **P005** | 57 | 0.4964 | 1 | **P005** | 20 | 0.8747 | 1 |
| **P006** | 164 | 0*** | 0*** | **P006** | 46 | 0.0473* | 0.7568 |
| **P007** | 17 | 0.8596 | 1 | **P007** | 9 | 0.9627 | 1 |
| **P008** | 54 | 0.0022** | 0.0336* | **P008** | 24 | 0.0277* | 0.4432 |
| **P009** | 99 | 0.0031** | 0.0512 | **P009** | 30 | 0.0185* | 0.296 |
| **P010** | 35 | 0.1397 | 1 | **P010** | 14 | 0.0814 | 1 |
| **P011** | 22 | 0.5329 | 1 | **P011** | 13 | 0.5298 | 1 |
| **P012** | 37 | 0.9555 | 1 | **P012** | 17 | 0.8547 | 1 |

**P*-value < 0.05, ***P*-value < 0.01, ****P*-value < 0.001, *N*: Sample size
