# Supplementary material for: Genetic diversity, relatedness and inbreeding of ranched and fragmented Cape buffalo populations in southern Africa
Source: PLoS One. 2020 Aug 14;15(8):e0236717. doi: 10.1371/journal.pone.0236717 (PMC7428177; doi:10.1371/journal.pone.0236717)
Supplement: Attachment — Submitted filename: Response to Reviewers.docx [file pone.0236717.s014.docx]

**Response to academic editor and reviewers**

We would like to thank the two reviewers for investing their time in reviewing our manuscript. We appreciate the constructive and detailed feedback provided by Reviewer 2 in particular. We also thank the academic editor, Professor Elissa Z. Cameron, for her time and efforts in handling this manuscript.

**Editor comments:**

Journal Requirements:

When submitting your revision, we need you to address these additional requirements:

1. Please ensure that your manuscript meets PLOS ONE's style requirements, including those for file naming. The PLOS ONE style templates can be found at <http://www.plosone.org/attachments/PLOSOne_formatting_sample_main_body.pdf> and <http://www.plosone.org/attachments/PLOSOne_formatting_sample_title_authors_affiliations.pdf>

>>>Response:

- Noted. Apologies for previous deviations from the style and/or file naming requirements.

2. In your Methods section, please provide additional location information of the study sites, including geographic coordinates for the data set if available.

>>>Response:

- Additional location information has been included, together with GPS coordinates of the National Parks. Private ranch locations were omitted as this may compromise the anonymity of the participating ranches. The location of the ranches was not important for the analyses and conclusions of the study, except to say that all ranches are located in South Africa. A statement to this effect has been included in the Methods section.
- It is unclear whether the geographic coordinates of individual samples from the National Parks were recorded upon collection and if so, these were not made available to us by the conservation authorities who performed the sampling. However, we felt that such fine scale GPS data were not needed to answer the questions of this study.

**Reviewer 1:**

Reviewer #1: This is a well-written manuscript which describes genetic variation in buffalo in private ranches in SA, as well as Graspan and Mokala NP populations which which were stocked through a breeding programme of Kruger NP buffalo, to establish disease-free populations with high genetic diversity. Populations on 12 private ranches in South Africa and Waterberg Plateau NP (Namibia) were measured, which established predominantly with founders from Addo, Graspan and Mokala, with some supplementation from zoos.

The question in my mind relates not to the quality of the work, which is sound, but to how broadly relevant it would be to readers of PLOSone. It might be better-suited to a journal such as the SA Journal of Wildlife Research.

>>>Response:

- We appreciate the reviewer’s comments on the manuscript. We refer to the stated scope of *PLOS ONE* (<https://journals.plos.org/plosone/s/journal-information#loc-scope>), which we believe our manuscript satisfies:

*“PLOS ONE* welcomes original research submissions from the natural sciences, medical research, engineering, as well as the related social sciences and humanities, including:

- **Primary research** that contributes to the base of scientific knowledge, including interdisciplinary, replication studies, and negative or null results.”

And (<https://journals.plos.org/plosone/static/publish>)”

“We evaluate research on scientific validity, strong methodology, and high ethical standards—not perceived significance.”

**Reviewer 2:**

Reviewer #2: This is a review of the manuscript PONE-D-20-00888 by de Jager et al. The manuscript presents a very large data set of microsatellite genotypes from South African buffalo populations representing national parks, semi-managed populations and private game ranches. The authors conduct a thorough set of analyses to infer whether genetic diversity is comparable in different classes of populations and to elucidate the genetic structure among populations.

While the analyses are generally competently carried out and an impressive number of complementary approaches are taken to verify various results, I think the manuscript suffers from trying to wring too much out of the data. The problem is that many of the analyses are based on assumptions that are blatantly violated in the special case of intensively managed or newly founded populations. It is also as a consequence too long, since the results can be boiled down to a very few bullet points, some of which are not new observations. Finally, there are problems and concerns with some of the analyses. I recommend the authors to reshape the manuscript focusing exclusively on 1) genetic diversity in the different populations, 2) inbreeding and 3) relatedness. These are the types of statistics that are informative and relevant for comparing the private and other populations. I therefore recommend that the authors submit a significantly abbreviated and modified version of the manuscript for review. My recommendations are provided in detail below.

>>>Response:

- We thank the reviewer for these suggestions to focus the content and message of the manuscript. We have taken these comments on board and focused the manuscript on four analyses: 1) Genetic diversity, 2) Relatedness, 3) Inbreeding and 4) Population differentiation. We provide more detailed responses to the reviewer’s comments below.

1) The Abstract is too long (although just within the journal limits) and contains several items of “Introduction” nature, such as detailed background about the provenance of each population which cannot be readily assimilated by the reader at this early point.

>>>Response:

- The abstract has been shortened by removing the background of the provenance of each population. New results about relatedness and inbreeding were also incorporated into the abstract.

2) The analyses presented in the section Population structure from L192 onwards are in my opinion not relevant, except perhaps the Fst calculations. The Structure analyses are problematic because the basic assumptions of the method are clearly violated. Structure assumes that K ancestral populations exist, from which the samples have their ancestry and which behaves in other respects like a WF population. This is clearly not the case in the complex network of the sampled populations, in which reticulated gene flow, founder events and in some cases selective breeding takes place. The authors go through considerable and commendable efforts in these analyses, but in my opinion it is very doubtful whether the results are meaningful. This is also reflected in the difficulty in establishing the “correct” K. At the very least the Structure plots are impossible to interpret. For example, why would both AENP and GNP/MNP contain admixture from each other? Why (if the authors’ speculation that the green cluster at K3 corresponds to an East African contribution) would GNP and MNP also carry this signal, albeit to a smaller extent? The authors should in my opinion either i) remove these analyses, ii) move them to a Supplementary document and significantly tone down the conclusions based on them or iii) convince me that these analyses tell us anything useful.

>>>Response:

- We do not entirely agree with the reviewer’s comments on the population structure analyses. While we agree that the Fst calculations are relevant, particularly for comparative purposes to previous (and future) studies, we think the Structure analyses also contribute some useful information/results. At the very least, it provides genetic support (for the first time) for the reported origin of buffalo on private ranches in South Africa, namely Addo and Kruger at least, and yes, a third gene pool unsampled in this study.
- We heeded the reviewer’s comments about the difficulty in establishing the “correct” K and removed all the Structure analyses except for the full data set and the relatives removed data set. While we wanted to show thoroughness in our exploration of the data and genetic structure, we lament that this came across as a difficulty in establishing the “correct” K, as this was not the intention. Thus, we focused the analysis on just the removal of relatives and the full data set. We justify our use of the “relatives removed” data set by showing that in this data set all locus pairs are in linkage equilibrium and all populations are in Hardy-Weinberg equilibrium (S7 Table and S8 Table), which are the assumptions made about the underlying data by Structure [1]. We concede that the full data set did not entirely conform to these assumptions. However, we are confident that K = 3 is the most accurate K we can estimate with this data set.
- Consequently, we have retained this analysis in the manuscript, but have moved the majority of the methods describing this analysis to S1 Appendix and moved the results to the supplementary information (S2 Fig, S3 Fig, S4 Fig – the DAPC analysis). We have cut down the discussion of the structure results substantially (L467-490). Furthermore, we provide explanations for the features of the assignment plots highlighted by the reviewer. We suggest what appears to be low levels of admixture between AENP and GNP-MNP is most likely shared ancestral polymorphism (L473) and not recent gene flow, particularly since this signal is very low at K = 3. Furthermore, we provide an explanation as to why GNP-MNP would contain a signal from the third cluster (perhaps East Africa), but also tone down the conviction with which we state this to reflect the possibility that this signal might be of proper East African origin, but is at least of non-South African origin (L483-490). We think this additional support strengthens our argument that private ranches consist of three gene pools and does not weaken this argument.
- We hope that the adjustments we made to the structure analyses and conclusions convince the reviewer of their robustness and provides enough support to include these analyses in the manuscript.

3) The estimation of the effective population size also does not convince me. Although I do not know the details of the LD based method, I doubt that the estimated Ne’s tell us anything useful about these populations. They all range within very similar values. The actual observed diversity statistics Ar and het are in my opinion more informative, again because it is doubtful whether these populations behave in any way in accordance with the underlying assumption of any population genetic model.

>>>Response:

- We note this concern and have significantly reduced the presentation of these results (moved to supplementary information, S1 Fig) and the discussion of Ne. We also included a statement (L406) reflecting the reviewer’s concern about the violation of population genetics models of the private ranches in particular, and we agree with the reviewer in this respect. We have, however, retained the discussion of Ne for the national parks, as we think it is a valuable analysis for these unmanaged, or semi-managed populations, particularly for comparisons to previous (and future) studies.

4) As above, the bottleneck estimation analysis is not very relevant. Due to the complex history and patchy ancestry of most populations (at least the private ranch populations), it is highly dubious whether we could reasonably expect a population genetically determined equilibrium correspondence between Ar and het. I would say that in all populations but AENP, this test is not meaningful.

>>>Response:

- We agree with the reviewer on this point and have removed the bottleneck estimation analysis and corresponding discussion.

5) I would have liked some further discussion about the breeding practice on the private ranches, and how this relates to the observed statistics. For example I would expect very high relatedness in the private ranches, given that all or most individuals probably share their father. Kinship coefficients are not discussed in the paper, although they were estimated as part of the downsampling procedure in the Structure analysis. I would say that a different relatedness pattern in farms relative to wild populations is one of the most obvious differences to look for, and the study should have this as one of the main aims. In fact, kinship coefficient distribution could be highly informative regarding the breeding practice in private and wild populations. For the same reason I think it quite surprising that private farms do not show any (substantial) difference in either Ne, het or Ar. The only reason I can think of is that wild buffalo populations approximate a similar mating system as that on the private ranches, but I don’t think this is supported by data on wild buffalo social structure.

>>>Response:

- We appreciate this comment, as we think the analyses that we conducted to address it has added a lot of value to the manuscript. We have included substantial analyses of relatedness and inbreeding (Methods: L181-204, Results: L251-293, incl. Figs 2, 3 and 4). The discussions of these results are scattered throughout the Discussion, but the main part is from L410-439. The discussions of these results had to be kept at high level, as information of the precise breeding protocols on each private ranch was not available for this study. We did, however, include a fine-scale study such as that as a future perspective.
- While one might expect a statistical significance test of a difference in mean relatedness between localities, the L-shaped distributions of pairwise relatedness values meant that the usual statistical tests for comparing distributions were not necessarily applicable here [2]. Instead, we compared the shape and tails of the distributions, which we think were more informative about the distributions of relatedness than just the mean (although we do report the mean as well).
- Similarly, with the distributions of individual inbreeding estimates, we think that the actual value of the mean is what was important, as the magnitude of this value has direct biological consequences, whereas measures of statistical differences between the means were not necessarily as informative. Particularly because most populations had low inbreeding.

6) Why did the authors not include some samples from eastern African populations, if they specifically want to assess whether there is East African ancestry in some populations? Such samples exist and are relatively abundant. There are even published microsat data sets, presumably (although I did not check) using the same microsatellite markers.

>>>Response:

- Unfortunately, we did not have access to East African samples during this study (and at present also do not have access to such samples).
- It was not an original aim of the study to assess whether there is East African ancestry in some populations, but it rather came about as an after-the-fact potential explanation, once we found K = 3 was the optimal K. However, we concede that we subsequently probably gave this result more importance than was required in the context of the whole study.
- We considered using published microsatellite data sets where a few loci do overlap with those in this study. However, we decided against this approach, because one of the major pitfalls of microsatellites as genetic markers is the difficulty to combine data sets of separate studies, or even the same samples genotyped in different labs. This is because there are many factors that can influence the scoring of alleles, such as the sequencing machine used, the polymer used within the capillaries of the machine, the reading/scoring rules such as binning, allele naming, how stutter peaks are dealt with and human error in scoring alleles. Therefore, it is difficult to reconcile microsatellite genotype data sets of different studies on the same species or even populations, unless the raw data is available for re-scoring. We did not want to introduce this uncertainty into the current study and so decided against using published data sets of East African buffalo here. However, perhaps an opportunity exists for a separate study to properly evaluate whether combining buffalo microsatellite data sets is feasible in this case.

7) I did not see the authors declare any conflict of interest. It needs to be evaluated how the mixing of commercial genotyping paid for by the private ranches has influenced any study decision.

>>>Response:

- We acknowledge this concern and we can honestly state that the private ranches had no input into the study design or conclusions, and all agreed to participate in the study regardless of the outcome, provided their identities remained anonymous. Therefore, we declared no conflict of interest. However, we can include the above statement in the conflict of interest section.

**References**

1.	Pritchard JK, Stephens M, Donnelly P. Inference of population structure using multilocus genotype data. Genetics. 2000;155(2):945-59. PubMed PMID: PMC1461096.

2.	Bradley JV. The insidious L-shaped distribution. Bulletin of the Psychonomic Society. 1982;20(2):85-8. doi: 10.3758/BF03330089.
