# Supplementary material for: Genetic diversity, relatedness and inbreeding of ranched and fragmented Cape buffalo populations in southern Africa
Source: PLoS One. 2020 Aug 14;15(8):e0236717. doi: 10.1371/journal.pone.0236717 (PMC7428177; doi:10.1371/journal.pone.0236717)
Supplement: Attachment — Submitted filename: Response to Reviewers.docx [file pone.0236717.s015.docx]

**Response to reviewers**

We would like to thank Reviewer 3 for their constructive comments on the manuscript. We believe the changes we implemented further improves the manuscript. Please see our responses below and note that all line numbers given in our responses refer to lines in the document with track changes (“Revised Manuscript with Track Changes.docx”).

**Reviewer 3**

The manuscript is very clear in its objectives and the authors properly investigated their question using appropriate statistical tests. Even if I wasn’t personally involved in the first review process, I can see that it was already greatly improved. Results are also properly presented. I feel like the research study is worth being published in PloS One, both considering the investigated number of specimens/ DNA markers and the research question. However, I have some comments which I would like to be addressed first.

**Material and methods:**

- large banks of tissue and blood from wildlife do exist in southern Africa, collected and preserved since decades. The authors are not specifying anything about the collection timing of the involved samples. Were the specimens sampled in a fair limited time period? And is this time period recent and comparable between populations? Further information regarding this point are essential to check if the authors properly interpreted their results. This could greatly impact the discussion.

Response:

**>>>** Sampling timing has been added to the manuscript (L107-109 and Table 1). All samples were collected within one buffalo generation (2008 – 2015) and 99% of the samples were collected between 2011 and 2015. We think this time period is recent enough, since only one to two buffalo generations have passed between sample collection and the writing of this manuscript. There is considerable overlap and/or few years difference regarding the timing of collection between populations (Table 1). Considering all the above, we are of the opinion that the collection timing does not have an impact on the discussion. We wrote the manuscript with this knowledge in mind but should have shared this information with the reader and thus thank the reviewer for pointing it out.

- why I fully understand to respect the private game ranches anonymity, I would still like that the authors present some extra numbers. Especially, the current Nc and the year when the ranches acquired their first buffalo on their property are in my opinion important for the discussion (considering the fairly long generation time (5-7 years) of buffaloes).

Response:

**>>>** We understand that the current Nc of private ranches would be interesting, particularly in the discussion of Ne. However, Reviewer 2 (in the previous round) expressed reservations about the Ne analysis and its discussion for the private ranches, which we subsequently toned down and shortened significantly, with a warning to interpret with caution. We do not think that Nc information would change this, as it does not change the nature of private ranch populations, which was the basis of Reviewer 2’s reservations. Furthermore, we unfortunately do not have the census data at hand for each private ranch and it may take months, or longer, to obtain this information from the owners, if it is recorded. Thus, on balance, we do not think that adding the census information would add substantial value to this study.

The date at which buffalo were first acquired by the various ranches may assist in interpreting the observed genetic diversity statistics, but considering the dynamic nature of buffalo populations on private ranches (buying and selling of buffalo), the year of establishment alone would, in our opinion, not be sufficient to properly interpret the genetic diversity in the manner we think the reviewer is suggesting. The lack of knowledge about the individual history of each private ranch is why we kept our discussion and analyses at a fairly high level, as we could not dive into the details of each ranch. We believe that it is beyond the scope of this study, but that it should rather be the focus of a separate study detailing more precisely how the individual histories of each private ranch (if this information can be obtained) affects the genetic diversity and relatedness of buffalo on each ranch.

- L144: you refer to “KNP breeding group”. What does this refer to? Wild herds or do they have kind of isolated groups of individuals in the NP from where they select the individuals which will be translocated?

Response:

**>>>** The breeding group consisted of ~140 buffalo cows and 10 bulls mainly from northern KNP (originally wild herds), that were contained in a fenced-off camp in the NP. This information has been added to the manuscript (L146 – 148).

**Results:**

- L220: “seventh largest sample size”: is this referring to all south African buffalo populations or the one sampled in your study?

Response:

**>>>** In the study. An edit was made to reflect this (L228).

- L311: so about 10% were removed, can you provide some numbers?

Response:

**>>>** The sample sizes before and after removal of relatives are provided in S7 Table (referenced a couple of sentences later). A line was also added to S1 Appendix (supplementary methods) after describing how relatives were removed to direct the reader to S7 Table for sample sizes before and after removal of relatives. The proportion removed varies for each sampling locality, depending on the relatedness levels observed.

- Bonferroni corrections: standard or sequential?

Response:

**>>>** Standard Bonferroni corrections were performed. This clarification was added to S1 Appendix, where the HWE analysis is described.

Discussion

- both considering the large number of private alleles and the STRUCTURE results, I understand the proposed hypothesis: an origin from an unsampled population. Why, however, focus on East Africa? What do you actually understand by East Africa? Something that wasn’t discussed is the fact that in southern African buffalo populations, three population clusters based on microsatellite genotypes were identified in Smitz et al. 2014. Considering that translocation is common practice in southern Africa, both between NP and ranches (and between them), could your signal not originate from the unsampled southern cluster (North Zimbabwe, Botswana)? Especially, a lot of buffalo from Hwange were translocated since decades, as this was also a disease free buffalo stock...

Response:

**>>>** The reviewer makes a good point here. We now define our understanding of East Africa in our discussion. Furthermore, we explicitly include in our discussion the possibility that the third signal may be from the northern cluster (North Zimbabwe, Botswana; the southern cluster is Hluhluwe-iMfolozi Park) as defined by Smitz et al. 2014. We previously referred to this possibility using the term “non-South African origin” but we now explicitly refer to the clusters identified by Smitz et al. 2014 as suggested by the reviewer, which we think improves the discussion. (L507-535). We also adjust the population history of the private ranches in the Methods section to reflect this other potential source (L169, L172).

[The reason we focused on East Africa is because of the East African origin of a few buffalo introduced into WPP (most likely Kenya or Tanzania – this is mentioned in the population history section of the methods), which also has the signal from the third gene pool. Additionally, some private ranches have introduced East African buffalo to their properties and advertise the fact they are breeding with these buffalo, although, admittedly, we do not know what their definition of East Africa is. East Africa for us is defined by the southern border of Tanzania. Thus, countries north/north-east of Tanzania are included, but mainly Tanzania and Kenya. Private buffalo owners may each be working under a different definition of East Africa, which opens this term up for interpretation and complicates genetics conclusion].

- statements L340 and L489: ok but important to specify that a re-evaluation will be needed when required. My point is that buffaloes have retained a great part of their historical genetic diversity – still recorded now, even after the severe Nc loss linked to the diseases outbreaks (multiple studies stating this). However, impact of the fragmentation of its habitat – which is quite “recent”, is not yet genetically measurable. Probably because not enough generations have elapsed since fragmentation has started. Also, its a vice versa situation. For now, if there is no external genetic inputs on private ranches, you also expect a decrease of the genetic diversity in the future. So it will highly depend on the management practices. Are some general long-term guidelines or regulations existing for the management of wildlife in private ranches in southern Africa?

Response:

**>>>** Yes, we agree that it is important to monitor genetic diversity of these populations. We have included a statement to highlight this important point (L557-559). The private ranches comment is addressed in the response to the next comment.

- Conclusion: To make such statements, you should consider the time in generations which elapsed since the private ranching practices have started to develop in southern Africa (info needed per ranches in the M&M). I would also suggest to be much more cautious when stating that genetic diversity etc. were not affected by the breeding strategies employed (L492). Not yet!

Response:

**>>>** We amended our conclusion related to the private ranches, thereby addressing this comment and the previous comment (L561-565). Since we do not have (and did not include) the date of establishment of buffalo populations on various private ranches, we made a more general cautionary statement on the importance of active management of buffalo on private ranches. Presently, there seems to be no unified regulations that exist for the management of wildlife on private ranches in southern Africa. We note this on L578-581.

- L373: careful with this statement, increased genetic diversity is not always beneficial: outbreeding, loss of local adaptations ... Why for example does the Eastern African buffalo males have longer horns? I guess it is not plasticity or there would be no point in translocating individuals from East to South.

Response:

**>>>** A qualifying statement has been added here (L386-388). Outbreeding depression/loss of local adaptation is also addressed on L490-495 in relation to genetic supplementation of AENP.

[This comment relates to WPP, which is a population established by Namibian authorities with buffalo from AENP and other sources. Thus, local adaptation has likely not yet occurred here and the probability of outbreeding depression is low between populations within the Cape buffalo subspecies in general, if one considers the guidelines suggested by Frankham et al. (1)].

- L378: see the importance to provide the Nc of the private ranches. Number of sampled specimens is not equivalent.

Response:

**>>>** We added an extension of this explanation (L393-394). The point made in this line refers to the fact that the sample size from P007 is small (not that the population is small), thereby implying that this sample size may not be as statistically robust as the sample sizes from the other localities, which then led to the significantly negative *F*_IS_ value. It is an explanation of a statistical nature rather than a biological one. Whether the samples adequately represent the population on this ranch, depending on the census size, is not what the statement was addressing.

- relatedness discussion: has to be re-evaluated in regard of the collection timing. Also, consider discussing the impact of the migration of only a few individuals per generation on the genetic structure of a population- literature about this aspect exist.

Response:

**>>>** As stated in response to the reviewer’s first comment, we are of the opinion that the collection timing does not have a significant influence, if any, on the discussion, given that all samples were collected in the space of one buffalo generation. The relatedness discussion was, however, restructured and amended to consider the effect of migrants on relatedness distributions- an explanation that we had overlooked. L429-444.

- L445: an important point is the use of FST as differentiation indices. This is tricky, especially when comparing different subspecies. FST is not a measure of “similarity”, it’s based on allelic frequencies. For example: if two populations are fixed for alternative alleles, then pairwise FST = 1. But if you have two populations, one with 50% aa and 50% bb, the other with 50% cc and 50% dd, then their pairwise FST = 0.33. Are the two last population genotypically closer to each other than the two first populations?

Response:

**>>>** We agree with the reviewer on this point and we are aware of this fact about *F*_ST_. We used *F*_ST_ for differentiation for compatibility with historical studies where *D*_JOST_ (a true differentiation measure) was not calculated. We thus also report *D*_JOST_, because it actually estimates differentiation. We added to the manuscript a correlation test between *F*_ST_ and *D*_JOST_ and show significant and strong correlation between the two measures (Spearman’s *rho* = 0.90, *p*-value < 2.2e^-16^). We think this adds some rigour to the use of *F*_ST_ as a proxy for differentiation for comparisons with previous studies, which we think is important to include in the discussion. This analysis is reported in the methods (L212-215), the results (L301-302) and the discussion (L467-469).

**General minor:**

- provide an access to your database – dryad or as supp material

Response:

**>>>** There should have been a link to the data set on Dryad. We thought we had provided it on the online submission system. We will check with the editor to ensure that the link is included in the final manuscript.

- check number of decimals you are using across the manuscript, ex. Line 249, 444.

Response:

**>>>** Thank you for pointing this out. We have checked and made sure we use the same number of decimals within each metric.

- “P” or “p” -value, homogenize

Response:

**>>>** Done.

- line 295-296: numbers are not essential, the most important is that they are not significant.

Response:

**>>>** Noted. Adjustments were made to these lines (L306-308).

- line 299: how did you calculated the average? Did you included the non significant estimates?

Response:

**>>>** All instances of “average” was replaced with the term “mean”, which is the most accurate term to use in this case, since it inherently provides information on how the “average” was calculated. Non-significant estimates were included, yes. The vast majority of pairwise estimates were significant.

**References**

1.	Frankham R, Ballou JD, Eldridge MDB, Lacy RC, Ralls K, Dudash MR, et al. Predicting the probability of outbreeding depression. Conserv Biol. 2011;25(3):465-75. doi: 10.1111/j.1523-1739.2011.01662.x.
